# TP53 R273C Mutation Is Associated With Poor Prognosis in LGG Patients

**DOI:** 10.3389/fgene.2022.720651

**Published:** 2022-03-11

**Authors:** Jian Zhang, Minglu Liu, Yujie Fang, Jinlong Li, Yin Chen, Shunchang Jiao

**Affiliations:** ^1^ School of Medicine, Nankai University, Tianjin, China; ^2^ Department of Oncology, Oncology Laboratory, General Hospital of Chinese PLA, Beijing, China; ^3^ Beijing DCTY Biotech Co., LTD, Beijing, China

**Keywords:** TP53 R273C mutation, prognosis, immune infiltration, tumor mutation burden, low-grade glioma

## Abstract

**Purpose:** With the progress of cancer immunotherapy, hotspot mutations of common oncogenes and tumor suppressors are becoming new potential therapeutic targets. TP53 R273C mutation is one of the hotspot mutations of TP53, and it has a higher frequency in low-grade glioma (LGG). However, the function of this mutation and its prognostic significance in LGG are not still clear.

**Methods:** To address this question, RNA sequencing, clinical, and SNP data of LGG patients from the TCGA database were downloaded. The Kaplan–Meier (KM) method was used for survival analysis. Immune cell populations in this cohort were assessed via the MCP counter and CIBERSORT. DNA damage/repair scores were calculated by GSVA analysis. WGCNA was conducted to identify genes related to TMB.

**Results:** In the context of IDH1/2 mutation, LGG patients with TP53 R273C mutation had worse prognosis than other mutation types and wild types. This conclusion is still valid in LGG patients who had received chemotherapy or radiotherapy. Considering the 1p19q codeletion status, it was found that patients with both R273C mutation and 1p19q non-codeletion had the worst prognosis. Further analysis showed that LGG patients with TP53 R273C mutation had higher M2 macrophage infiltration and tumor mutation burden (TMB) than that of TP53 wild-type LGG patients, and higher TMB indicates poor prognosis in LGG patients. Furthermore, we identified genes which could be associated with higher M2 macrophage infiltration and TMB in LGG patients with TP53 R273C mutation.

**Conclusion:** The study indicates that TP53 R273C mutation is very likely oncogenic and may be used as an indicator of the prognosis of LGG.

## Introduction

Gliomas are the most common primary malignant brain tumors and can be classified into four grades (grades I–IV) according to the WHO grading system (Louis et al., 2016). Low-grade gliomas (LGGs; WHO grade II and III gliomas) (Cancer Genome Atlas Research et al., 2015) are less aggressive than grade V glioma, that is, glioblastoma (GBM). Surgical resection combined with chemoradiotherapy is the standard treatment (Weller et al., 2017). However, LGG patients have highly diverse clinical outcomes. A subset of these gliomas will progress to GBM within months, whereas others remain stable for a long time (van den Bent et al., 2013).

Although the histopathological classification of LGGs is widely recognized, it cannot adequately predict survival. Therefore, clinicians tend to rely on genetic classifications to guide treatment. Mutated genes such as IDH1, IDH2, TP53, EGFR, and ATRX, 1p/19q codeletion, and MGMT promoter methylation are well-recognized prognostic factors for LGG patients. Patients with LGGs carrying isocitrate dehydrogenase (IDH1/2) mutation live significantly longer than those with IDH wide tumors. In the context of IDH mutation, 1p/19q codeletion represents a favorable result compared with 1p/19q non-codeletion (Louis et al., 2016). However, these genetic factors fail to evaluate survival outcomes accurately. Both LGG patients with 1p/19q codeletion and non-codeletion are heterogenous, which suggests that additional factors may help in determining prognosis.

TP53 is one of the most frequently mutated genes in LGGs. TP53 mutation closely overlaps with mutations in ATRX and 1p/19q non-codeletion (Cancer Genome Atlas Research et al., 2015). Our present work has shown that the frequency of TP53 R273C mutation is significantly higher than that of other missense mutations of TP53 in LGGs, which is consistent with a previous study (Petitjean et al., 2007). Obviously, the R273C mutant may play a vital role in the survival of LGGs. As a transcript factor, wild-type TP53 regulates gene expression by directly binding to DNA in a sequence-specific manner. The R273 residue in TP53 contacts DNA directly, so the R273 mutant is probably highly oncogenic in theory. However, unlike the R273H mutant, which is a well-recognized oncogenic mutant, the function of the R273C mutant is controversial. The TP53 R273C mutant can significantly increase the expression of HER2 in the C33A cervical cancer cell line (Roman-Rosales et al., 2018). TP53 with R273C mutation has failed binding to the DNA repair gene RAD51 in breast cancer lines (Rasti and Azimi 2015). However, liver cancer cells Hep3B expressing the p53 mutant, R248Q, but not R273C, displayed cross-resistance to doxorubicin and paclitaxel (Chan and Lung 2004). The TP53 R273C mutant can even promote the expression of the tumor suppressor gene retinoic acid receptor beta when HPV 16 is present (Zhang et al., 2017). So far, the function of R273C mutation in TP53 has not been well identified. In this study, we analyzed the prognostic significance of TP53 R273C mutation in the TCGA–LGG cohort, which had a larger sample size. In addition, we also explained this change by analyzing the tumor mutation burden (TMB) and immune cell infiltration. Our study suggests that TP53 R273C mutation is an unfavorable prognostic biomarker for LGG patients, and this may be associated with a higher TMB value.

## Methods

### Patients and Data Sets

RNA-Seq count data (*n* = 529) and somatic mutation data (*n* = 506) were downloaded from the TCGA data portal (https://gdc-portal.nci.nih.gov/). Clinical information (*n* = 516) was downloaded from cBioPortal (https://www.cbioportal.org/). After excluding recurrent LGGs and data integration, 502 LGGs with survival data were included in this study. 1p/19q codeletion data were obtained from publication (Hu et al., 2017). Methylation data was downloaded from https://biit.cs.ut.ee/methsurv/data/LGG_meth.RData.

### The Estimation of Tumor Mutation Burden and Immune Environment

TMB was defined as the total somatic nonsynonymous mutation counts in coding regions per million bases. In our study, we calculated the mutation frequency with the number of variants/the length of exons (38 million) for each sample via Perl scripts which is based on the JAVA8 platform (Zhang et al., 2019). R package software MCP-counter (Becht et al., 2016) was employed to analyze the mRNA expression matrix to produce the absolute abundance scores for eight major immune cell types (CD3^+^ T cells, CD8^+^ T cells, cytotoxic lymphocytes, natural killer cells, B lymphocytes, monocytic lineage cells, myeloid dendritic cells, and neutrophils), endothelial cells, and fibroblasts. The relative abundance scores of 22 immune cells in all tumor samples were calculated by using CIBERSORT (Newman et al., 2015). To quantify the DNA damage/repair pathway enrichment score across samples, GSVA (Gene Set Variation Analysis) was performed using the R package GSVA (v.1.32).

### Cell Culture and Stable Cell Line Construction

SW1088, a kind of grade III glioma cell line, was obtained from COBIOER and authenticated by STR DNA profiling. SW1088 cells were grown in high-glucose the Dulbecco’s modified Eagle’s medium (DMEM) supplemented with 10% fetal bovine serum and cultured at 37° C in a 5% CO_2_ humidified atmosphere.

To knock out the TP53 gene from SW1088 cells, gRNA-targeting exon 8 of the human TP53 gene was designed using CRISPOR software (www.crispor.tefor.net). The gRNA sequence is 5′-ACU​GGG​ACG​GAA​CAG​CUU​UG-3’. CRISPR/Cas9-based knockout was performed by electroporation of the Cas9/sgRNA (RNP) complex using the Celetrix electroporator. Briefly, 3 µg cas9 protein (GenScript, NLS-Cas9-NLS Nuclease) and 1.5 ug gRNA were added to 10 ul electroporation buffer and incubated for 10 min at 37° C. A total of 2 × 10^6^ SW1088 cells were collected and resuspended in 15 µl electroporation buffer. Then, the RNP complex was added to the cell suspension and mixed well. At last, the electroporation was carried out in a 20 µl tube under the condition of 400 V and 30 ms. A single-cell clone was picked by using the Sony Cell Sorter. Sanger sequencing and Western blotting were used to identify the TP53-knocked-out clone.

To generate TP53 R273C-expressing SW1088 cell lines (SW1088-TP53R273C), SW1088-TP53^KO^ cells were infected in the exponential growth phase with retrovirus-carrying TP53 R273C cDNA.

SW1088-Control was generated by infection with control retrovirus (the empty vector). The expression of the TP53 protein was detected by Western blotting. The monoclonal antibody for TP53 was purchased from Abcam (ab1101). These cells were used for experiments 1 week after transfection.

### Cell Apoptosis and Growth Assays

For apoptosis assay, the SW1088-TP53^KO^, SW1088-TP53^R273C^, and SW1088-Control (the empty vector) were plated at a seeding density of 2 × 10^5^ cells/well in 6-well plates. 20 h later, cells were treated with 500 μM temozolomide (TMZ) or irradiated with 10 Gy of X-rays. The control group was left untreated. Each group has two parallel wells. 48 h later, cells were digested with trypsin without EDTA, then analyzed using flow cytometry after staining with AnnexinV-APC (Biolegend) and zombie violet (Biolegend).

Cell growth was assessed using CCK8 assay following manufacturer’s instructions. About 2 × 10^3^ cells (100 ul) were plated in a 96-well plate. The absorbance at 450 nm was measured to evaluate the cell viability at 0 h (12 h after cells were seeded), 24, 48, 72, and 96 h. CCK8 reagents (10 µl) were added to each well and incubated for 1 h at 37°C.

### Statistical Analysis and Plotting

Differential analysis of RNA sequencing read counts were analyzed using the edgeR package (version 3.26.8) (Robinson et al., 2010) with TMM normalization. Weighted gene correlation network analysis (WGCNA) was performed using the WGCNA R package (version 1.69) (Langfelder and Horvath 2008).

All statistical and bioinformatics analyses were performed with R (v3.6.2). For survival analysis, we used the R survival package. All the survival analyses were performed using Kaplan–Meier survival analyses. *p*-values were calculated from log-rank tests. All statistical tests were two-sided, with *p* values less than 0.05 considered statistically significant.

## Results

### R273C Is the Most Frequently Observed TP53 Mutant and Suggests Poor Prognosis in Lower Grade Glioma

**FIGURE 1:**
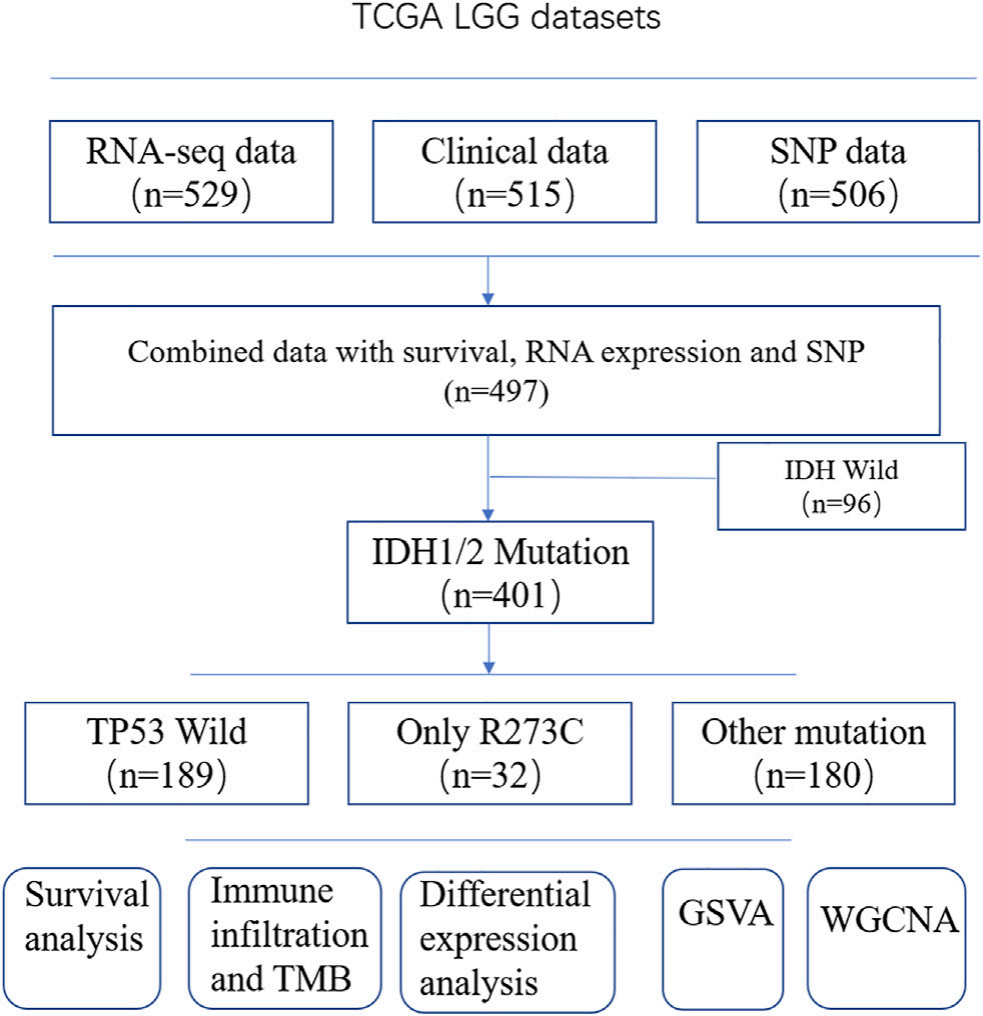
Study flowchart. Patients (*n* = 9) whose TP53 genes contain not only R273C mutation but also other mutations were included in the other mutation group.

**FIGURE 2:**
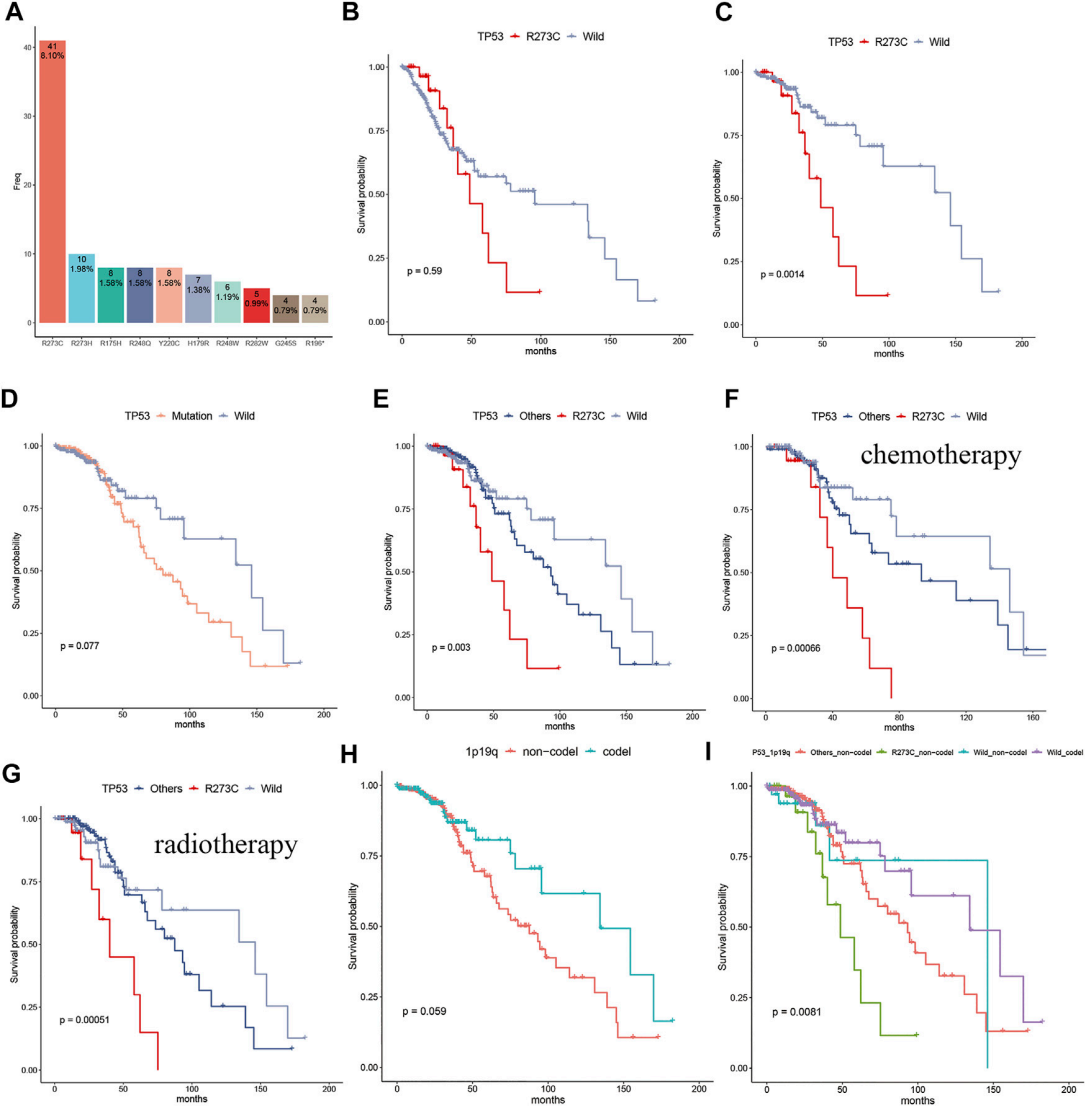
The frequency of the TP53 R273C mutant and its prognostic significance in LGG patients. **(A)** The bar plot showed the frequency of TP53 hotspot mutation in LGGs, and the R273C mutant had the highest mutation frequency, according for 8.1% in the whole LGGs. **(B)** Comparison of survival of the R273C (*n* = 32) and the TP53 wild groups (*n* = 269) in the whole LGG patients. **(C–I)** Comparison of survival in the LGG patients with IDH1/2 mutation. **(C)** R273C (*n* = 32) mutation vs. wild (*n* = 189). **(D)** mutation (*n* = 212) vs. wild (*n* = 189). **(E)** R273C (*n* = 32) vs. others (*n* = 180) vs. wild (*n* = 189). **(F)** R273C (*n* = 22) vs. others (*n* = 96) vs. wild (*n* = 96) in patients who received chemotherapy. **(G)** R273C (*n* = 22) vs. others (*n* = 119) vs. wild (*n* = 79) in patients who received radiotherapy. **(H)** 1p19q codel (*n* = 155) vs. 1p19q non-codel (*n* = 245). **(I)** others_non-codel (*n* = 175) vs. R273C non-codel (*n* = 31) vs. wild codel (*n* = 150) vs. wild non-codel (*n* = 39).

A brief flowchart of data processing is presented in Figure 1. All the Patients were pathologically and clinically diagnosed with LGG. The R273C mutant occurs in about 2.7% of all human cancers (Li et al., 2014) and about 8.1% (41/506) of LGGs in this study. The mutation frequency of TP53 R273C is significantly higher than other TP53 mutants in LGGs, as shown in Figure 2A. This is coincident with previous studies (Petitjean et al., 2007; Salnikova 2014). To date, the function of the R273C mutant has not been well identified, and conclusions from different studies are controversial. The prognostic roles of the TP53 R273C mutant in LGGs are still obscure. As the mutation frequency was higher, we reasoned that the R273C mutant was oncogenic in LGGs. To test our hypothesis, we examined the survival of the TP53 R273C and TP53 wild groups with Kaplan–Meier analysis. Unexpectedly, the survival difference between the R273C mutant and wild type in the whole LGG patients was not statistically significant (*p* = 0.59) (Figure 2B). This did not, however, preclude that other factors might contribute to patients’ survival. We first compared the expression level of TP53 between the two groups. However, no difference was observed (Supplementary Figure S1A). The heatmap of the mutation landscape and clinical information (Supplementary Figure S1B) showed that all the TP53 R273C mutations coexisted with IDH1/2 mutation, while the part of wild TP53 coexisted with IDH mutation, which was a recognized poor prognostic indicator for LGGs. We then compared the survival of the two groups in patients with IDH1/2 mutation. Figure 2C shows that the R273C mutant group had significantly worse overall survival (OS) than the wild-type group (*p* = 0.0014). Interestingly, the OS of the whole TP53 mutation group was not significantly worse than the TP53 wild-type group (Figure 2D), which indicated that not all the TP53 mutants had the same functional change. This is demonstrated in Figure 2E. The other groups, which contained all the other mutations except the R273C mutant, had worse OS than the wild group but had better OS than the R273C group. This trend was also found in patients who received chemotherapy or radiotherapy (Figures 2F,G). To further assess the value of TP53 R273C mutation as a prognostic marker, the 1p19q status and MGMT (O6-methylguanine-DNA methyltransferase) promoter methylation, both of which are well-recognized prognostic biomarkers for LGG patients, were considered in the survival analysis. Patients with 1p19q non-codeletion had worse OS than that that of patients with 1p19q codeletion (*p* = 0.059) (Figure 2H). When analyzed based on the 1p19q and TP53 mutation statuses, we found that patients with 1p19q non-codeletion and TP53 R273C mutation could be the worst patient populations with IDH1/2 mutation (Figure 2I). Although MGMT promoter methylation is a well-known prognostic biomarker, the methylation level was not related to prognosis in LGG patients with IDH1/2 mutation of the TCGA cohort (Supplementary Figure S2A). When analyzed together with the TP53 mutation status, patients with TP53 R273C still had the worst prognosis regardless of the MGMT promoter methylation level (Supplementary Figure S2B).

### Comparison of Clinical Characteristics and Immune Infiltration Among Different Lower Grade Glioma Subsets

**FIGURE 3:**
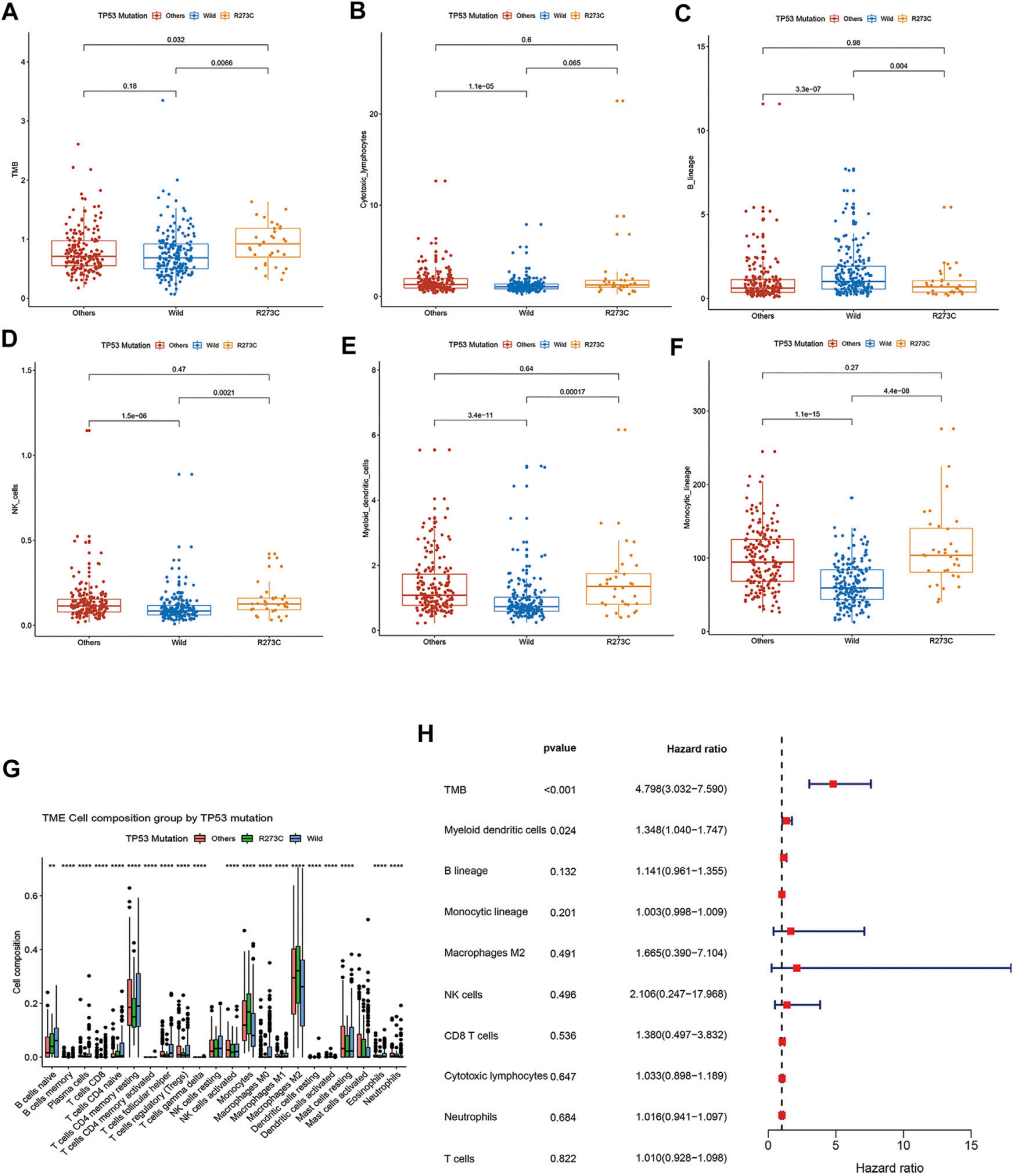
comparison of TMB and immune cell infiltration among different groups. **(A)** TMB, **(B)** cytotoxic lymphocytes, **(C)** NK cells, **(D)** B cells, **(E)** monocytic lineage cells, **(F)** myeloid dendritic cells, and **(G)** Immune cell component comparisons between TP53 mutation and wild-type groups analyzed by CIBERSORT. **(H)** Forest plot of univariate Cox proportional hazards regression analysis of overall survival.

Next, we analyzed the characteristics of the three groups. Both TP53 R273C mutation and other mutations were found predominantly in younger patients (Table1), which is consistent with the previous study (Salnikova 2014). There were no significant differences in gender and grade rates among the three groups (Table1). To investigate the profile of immune infiltration in different TP53 mutation subsets, CIBERSORT and MCP-counter were manipulated to estimate the abundances of immune cells infiltrating into LGGs. TMB and many infiltrating immune cells were found to be significantly heterogeneous in different LGG subsets. The TMB of the R273C group was significantly higher than that of other mutation and wild-type groups. Interestingly, we did not find any significant difference between other mutation and wild-type groups (Figure 3A). There was no difference in the abundance of T cells, CD8^+^ T cells, and neutrophils (Supplementary Figure S3A–C), and significant differences were observed in the number of cytotoxic lymphocytes, natural killer cells, B lymphocytes, monocytic lineage cells, and myeloid dendritic cells among the three groups (Figures 3B–F). Specifically, the R273C and other mutation groups had similar immune cell infiltration. Also, the immune cell infiltration of the whole TP53 mutation group was different from the wild-type group. Cytotoxic lymphocytes, natural killer cells, monocytic lineage cells, and myeloid dendritic cells showed significantly higher infiltration in the TP53 mutation group, while B cells showed significantly lower infiltration in the TP53 mutation group. Monocytic lineage was the major immune component of leukocyte infiltration in LGGs. Further analysis by CIBERSORT revealed that M2 macrophages were the predominant component in LGGs and showed different infiltration levels (Figure 3G). Of the nine cell populations and TMB, only TMB and myeloid dendritic cells were correlated to prognosis in univariate cox survival analysis (Figure 3H). A higher TMB predicts poor survival in LGG patients, which is consistent with the previous study (Yin et al., 2020). To identify the relationship between TP53 R273C mutation and immune checkpoint genes (ICGs), we downloaded the ICG gene list from recent literature (Fei-Fei Hu et al., 2021). The relationship was presented in the volcano plot (Supplementary Figure S4). Several HLA molecules and co-stimulatory molecules were significantly downregulated in the TP53 R273C mutation group.

**TABLE 1:** Comparison of clinical characteristics among TP53 mutation subsets in the IDH1/2 mutation background.

	Wild	Others	R273C	ALL	*p* Value
Age (mean ± SD)	45 ± 13	37 ± 10	37 ± 11	41 ± 12	0.000
Gender (male/female)	102/87	107/73	15/17	224/177	0.323
Grade (G2/G3)	107/82	88/91	20/12	215/185	0.360
Chemotherapy (no/yes)	82/96	67/96	10/22	159/214	0.120
Radiotherapy (no/yes)	92/85	40/129	8/24	140/238	0.000

Some patients’ clinical information was lost. The Student t test (Age) and χ^2^ test (gender, grade, chemotherapy, and radiotherapy) were used for statistical significance.

### TP53 R273C Mutant Can Modulate Immune Cell Infiltration Through Multiple Pathways

**FIGURE 4:**
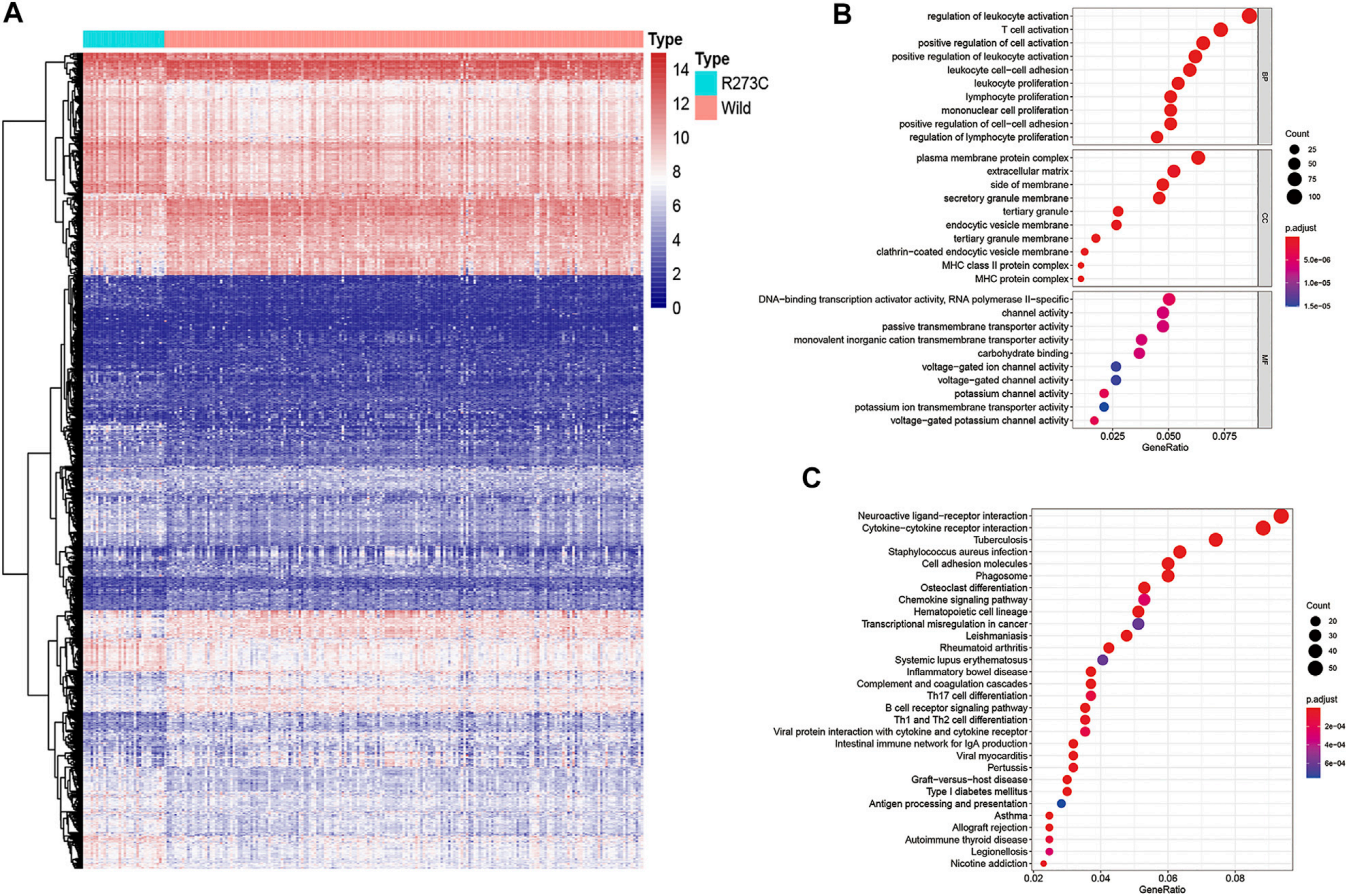
Heatmap and functional annotation of differentially expressed genes. **(A)** Heatmap of genes showing significant differential expressions (adjusted *p*-value < 0.05, |log FC|>1). We used log2 (TMM +1) for log-scale. **(B–C)** GO **(B)** and the KEGG **(C)** annotation of the genes that were significantly differentially expressed in both populations.

To clarify the potential mechanism of TMB and immune infiltration difference between TP53 R273C mutation and the wild-type group, we analyzed the gene expression between the two groups. To sum up, 778 genes were upregulated (≥2 fold), and 602 genes were downregulated (≥2 fold) in the R273C group (wild-type group as the reference, Figure 4A). Next, we conducted gene ontology (GO) enrichment analysis and the Kyoto Encyclopedia of Genes and Genomes (KEGG) pathway analysis with these differentially expressed genes (DEGs). The results showed that 785 GO terms were enriched for biological processes (BP), 81 GO terms were enriched for cellular components (CC), 74 GO terms were enriched for molecular functions (MF), and 54 KEGG pathways were enriched. Finally, the GO and KEGG enrichment bubble plots were made to show the top-10 entries of the GO or top-30 entries of the KEGG pathway analyses (Figures 4B,C). The enrichment GO terms were mainly involved in leucocyte activation and proliferation, which could in part account for the differential immune cell infiltration between the TP53 R273C mutation and wild groups. Since M2 macrophages were the main components in the immune environment of LGGs, we next investigated how TP53 R273C mutation resulted in higher M2 macrophage infiltration. Among the top-30 enriched KEGG pathways, the cytokine–cytokine receptor interaction and chemokine-signaling pathways may regulate M2 polarization. So, we merged the genes in the two pathways, and the unique 67 genes are shown in Table 2. Previous studies have demonstrated that the IL10-signaling (Cheng et al., 2018) and M-CSF/CSF1R pathways (Cuccarese et al., 2017) accounted for M2 polarization. In Table 2, IL10RA and CSF1R were upregulated in the R273C group.

**TABLE 2:** Genes in the cytokine–cytokine receptor interaction and chemokine signaling pathways.

BMP8B	CSF1R	DOCK2	IFNLR1	IL27	PIK3R5	TNFSF14
CCL1	CSF2RA	EBI3	IL10RA	IL2RB	PLCB2	TNFSF18
CCL13	CSF2RB	EDA2R	IL11RA	IL31RA	PLCG2	TNFSF8
CCL24	CSF3R	FASLG	IL12RB1	IL6	PRKCD	VAV1
CCR1	CX3CR1	GDF15	IL13RA1	JAK3	RAC2	WAS
CCR2	CXCL10	GDF7	IL15RA	LYN	SHC3	XCL2
CCR5	CXCL13	GNG12	IL16	MSTN	TGFB2	XCR1
CD4	CXCL17	GNG5	IL18	NCF1	TNFRSF11A	
CD70	CXCR1	GNGT2	IL1A	NGF	TNFRSF1B	
CNTF	CXCR2	HCK	IL1B	PIK3CG	TNFRSF8	

### Higher Tumor Mutation Burden Is Associated With DNA Damage/Repair Pathways

**FIGURE 5:**
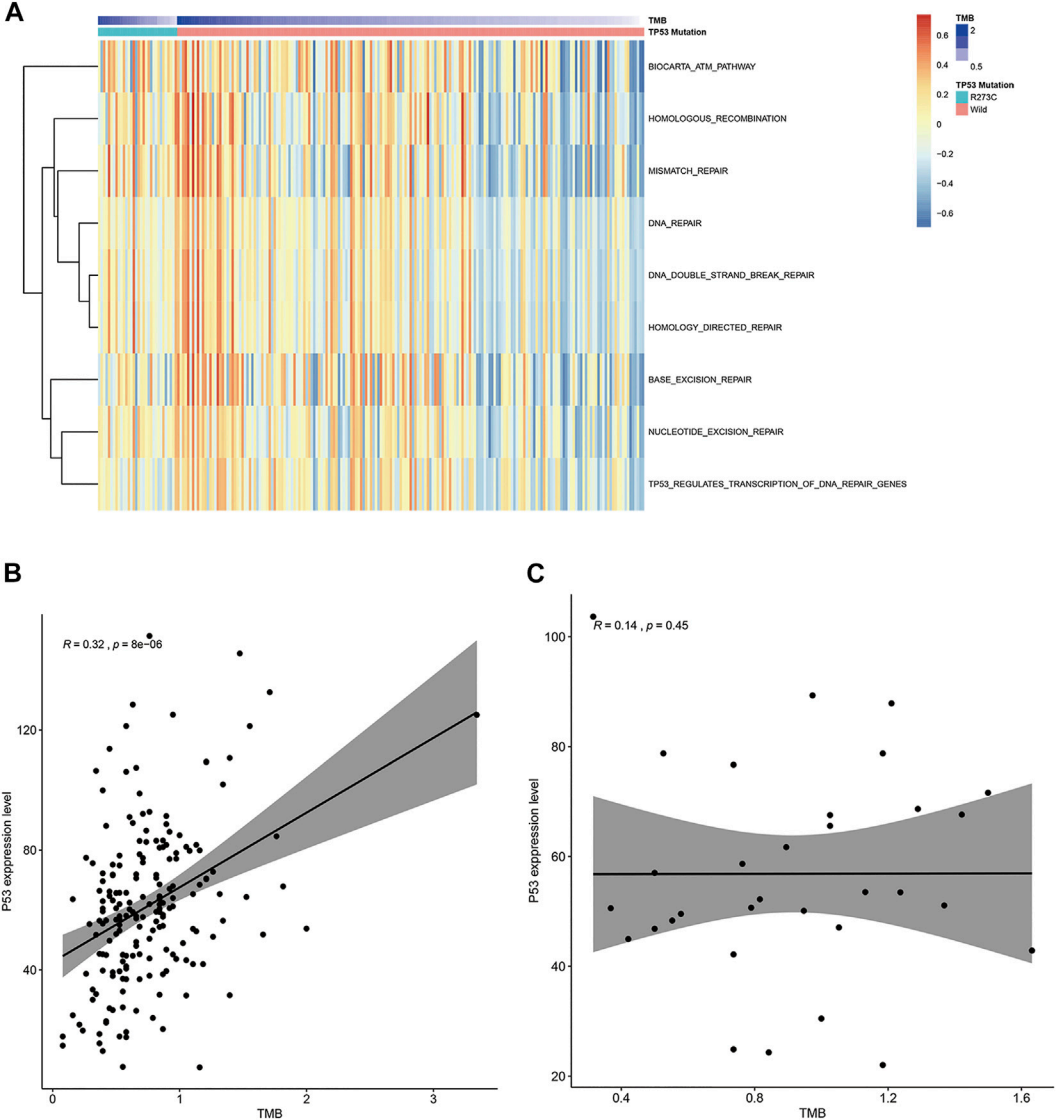
TMB and DNA damage/repair. **(A)** The association between TMB and DNA damage/repair pathways. **(B, C)** The relationship between TMB and the TP53 expression level in patients with wild-type TP53 **(B)** and the TP53 R273C mutant **(C)**; R, Spearman coefficient.

Some studies showed that TMB was associated with DNA damage/repair pathways. Tumor mutation means DNA damage, and a higher TMB means a higher DNA damage level. DNA damage will cause DNA repair, which needs cell cycle arrest, and TP53 is a key regulator of cell cycle arresting. In summary, a higher TMB induces the expression of wild-type TP53, then results in the arresting of the cell cycle, and induces DNA repair (Aubrey et al., 2018; Jiahao Hu et al., 2021). However, no correlation was observed between TMB and DNA damage/repair pathways in the above enrichment analysis. To further analyze this phenomenon, DNA damage/repair-related gene sets were downloaded from the Molecular Signatures Database (MSigDB; www.broadinstitute.org/gsea/msigdb). Then, GSVA scores for every sample were calculated and are shown in Figure 5A. It is obvious in Figure 5A that TMB was positively correlated at different levels with DNA damage/repair response scores of all the nine DNA damage/repair-related gene sets, and this seemed to be more obvious in the TP53 wild group. Another remarkable feature in this figure was that the strongest DNA damage-repair response occurred in patients with higher TMB and wild TP53. This indicated that wild TP53 played a crucial role in the DNA repair pathway. We further reasoned that TMB was positively related with the TP53 expression level in TP53 wild LGG patients, and this is demonstrated in Figure 5B (*R* = 0.32, *p* = 8e-06). No relation was found between TMB and the TP53 expression level in TP53 R273C LGG patients (Figure 5C).

### Potential Mechanism for the Higher Tumor Mutation Burden in the TP53 R273C Group

**FIGURE 6:**
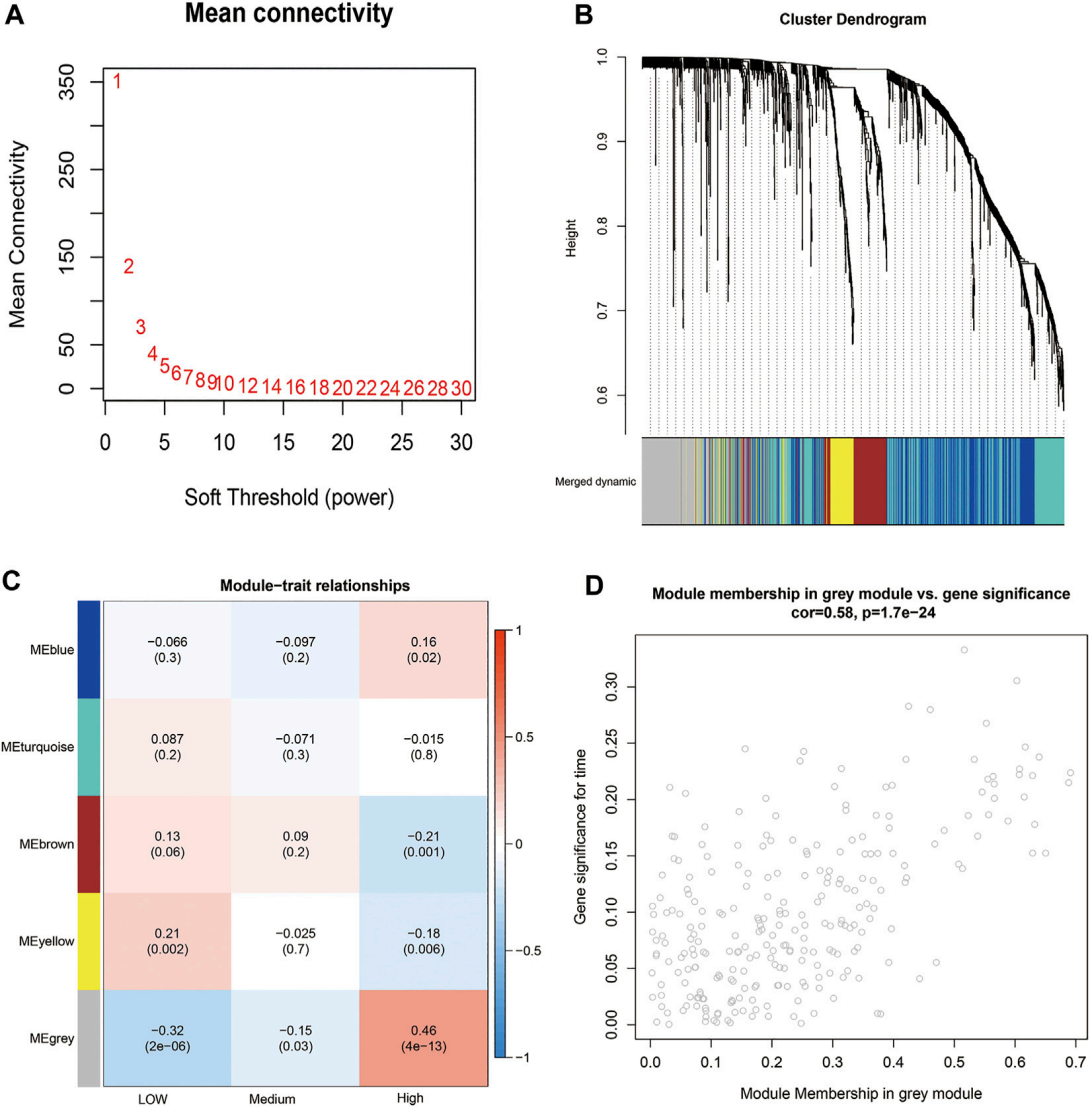
WGCNA. **(A)** Analysis of the mean connectivity for different soft-threshold powers. **(B)** Cluster dendrogram based on dynamic tree cut. **(C)** Module–trait relationship plot. Each row corresponds to a module and column to TMB. **(D)** Scatterplot of gene significance versus module membership for the grey module.

The mechanism of TP53 R273C mutation having higher TMB than the TP53 wild group has not been defined. We tried to determine which genes in the 1380 DEGs may affect TMB by using WGCNA. The co-expression network was constructed using LGG patients with the TP53 R273C mutant or wild group. Hierarchical clustering indicated that there was no outlier in the dataset (Supplementary Figure S5A). The power of *β* = 7 was selected as the soft threshold parameter to ensure a scale-free network (Figure 6A). Specifically, five co-expression modules were identified (Figure 6B). Then, we tested the relationship between each module and TMB. Patients were divided into low (*n* = 73), medium (*n* = 73), and high (*n* = 75) TMB based on TMB tertile distribution. The module eigengene of the grey module showed the highest association with TMB (Figure 6C). Next, we performed GO enrichment analysis based on 257 genes of this module using the “clusterProfiler” R package. In the GO analysis, terms related to morphogenesis and development were most prominent, such as skeletal system development, limb development, and appendage development (Supplementary Figure S5B). These findings revealed that genes from the identified grey module were mainly involved in cell differentiation.

Then, we identified 61 hub genes based on module membership >0.5 and gene significance >0.15 (Figure 6D). Interestingly, 13 out of 61 genes belong to the family of HOX genes (Table 3), and all of them were significantly upregulated in the TP53 R273C group.

**TABLE 3:** Hub genes of the grey module.

ABTB2	CYP1A1	GPR27	HOXA7	HSD17B6	OLFM4	SHISA6
ACSL6	DAO	GRIN3A	HOXA9	HTR2A	OR4N2	SIX1
ADARB2	DEPDC1	GUCY1A1	HOXC10	KCNIP2	OSR1	SLC24A4
ASPM	E2F2	HAND2	HOXC9	KIF2C	OSR2	SLC6A18
C14orf39	ENDOU	HKDC1	HOXD10	KIF6	P2RY1	SNORC
CALN1	EPB41	HOXA10	HOXD11	KIT	PDIA5	SPOCD1
CD300LG	EVX1	HOXA11	HOXD13	LHX1	RASSF2	VSTM2A
CFAP47	F5	HOXA5	HOXD8	MYOD1	SALL4	
CHL1	FOXD1	HOXA6	HOXD9	NKX1-2	SDS	

### TP53 R273C Mutation Contributes to the Resistance of SW1088 to Temozolomide and Irradiation

**FIGURE 7:**
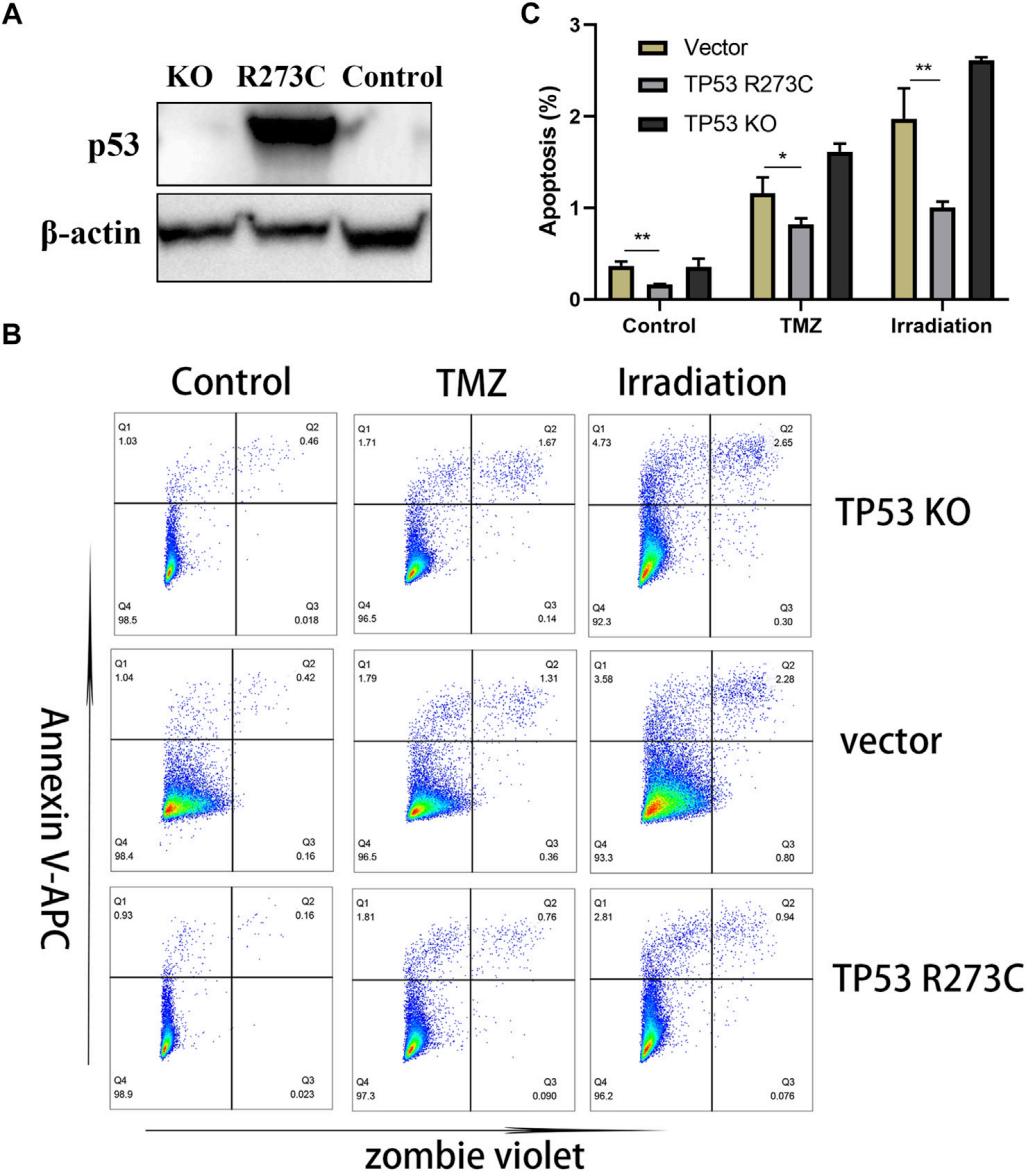
Western blotting and functional assays. **(A)** WB results of TP53 expression in SW1088-TP53^KO^, SW1088-TP53^R273C^, and SW1088-Control (the empty vector). **(B–C)** Flow detection of apoptosis. Statistical significance: * 0.05 < *p* < 0.01, ** 0.01 < *p* < 0.001 for the multiple *t*-test.

In our study, LGG patients with TP53 R273C mutation had a poorer prognosis than those with other TP53 mutations, and this trend was also found in patients who received chemotherapy or radiotherapy, which indicates a complete loss of function of TP53 R273C mutants or even a GOF mutation in LGG. To further prove that TP53 R273C mutation can promote resistance to chemotherapy and radiotherapy, we first knocked out TP53 of SW1088, a kind of LGG cell line. Then, polyclonal cell line SW1088-TP53^KO^ was obtained by single-cell sorting (Supplementary Figure S6A). To generate stable transfection cell lines, TP53 R273C cDNA was cloned into the lentivector pCDH -MSCV-MCS-T2A-GFP, respectively. Finally, we obtained the stable transfection cell lines SW1088-TP53^R273C^ and SW1088 vector control (Figure 7A). Then, cells were treated with temozolomide or irradiation. The result of apoptosis analysis by flow cytometry is shown in Figures 7B,C, and an example of gating strategy (FSC vs SSC) is shown in Supplementary Figure S6B. The rate of apoptosis was significantly lower in SW1088-TP53^R273C^ cells than that in SW1088-TP53^KO^ and SW1088-Control in all of the three treatment conditions. To explore the effects of TP53 R273C mutation on tumor proliferation, cell proliferation was determined by CCK8 assay. We found that the overexpression of the p53 R273C mutant did not affect SW1088 proliferation *in vitro* (Supplementary Figure S6C–D). Promotion of proliferation and resistance to apoptosis are two interrelated but distinct biological processes. The p53 R273C mutant may not only lose the function of wild-type p53 but also acquire the function of resistance to apoptosis *via* some potential mechanisms in SW1088.

## Discussion

Although the R273C mutation has a higher frequency in LGG, due to the limitation of the sample size and bioinformatics, the prognostic significance and function of this mutation in LGG are not yet clear. In this study, we used the TCGA LGG datasets, which have a large sample size, comprehensive clinical information, and RNAseq and SNP data, for in-depth analysis of this problem.

First, we found that in the context of IDH1/2 mutations, LGG patients with R273C mutation had worse prognosis than other mutation types and wild types. This conclusion was still valid in LGG patients who have received chemotherapy or radiotherapy, which was proved by the apoptosis experiment (Figures 7B,C). It showed that the LGGs with TP53 R273C mutation had a certain degree of resistance to radiotherapy and chemotherapy, which had several implications for the clinical work. In addition, taking into consideration the status of 1p19q codeletion, it was found that patients with both R273C mutation and 1p19q non-codeletion had the worst prognosis. This could be helpful for improving the existing prognostic evaluation system. It is important to note that although LGG patients with TP53 R273C mutation have a worse prognosis than other mutation groups, it cannot be considered that the prognosis of patients with TP53 R273C mutation is worse than that of all other mutation types. Because some mutations in other mutation groups were partial, loss-of-function mutations and others were well-recognized in oncogenic mutations (Stiewe and Haran 2018).

In addition, despite the large sample size in this study, there were not many patients with TP53 R273C mutation. Thus, some precautions should be taken on the above conclusions.

To illuminate the reasons for the poor prognosis of patients with TP53 R273C mutation, we compared the differences in immune cell infiltration and TMB between the mutation and the wild groups and found that the TP53 R273C group had more M2 macrophage infiltration and higher TMB. In recent years, the relationship between TP53 and the immune system is receiving increasing attention. TP53 mutation can affect the distribution and function of T cells, NK cells, and macrophages (Blagih et al., 2020a; Levine 2020). P53 deletion can promote myeloid-associated cytokines in PDAC cells, such as CCL11, CXCL1, CXCL5, CCL3, and M-CSF (Blagih et al., 2020b). In this study, IL10-signaling and M-CSF/CSF1R pathways may have been involved in M2 polarization.

Loss of TP53 DNA damage checkpoint activity, by somatic mutation, copy number loss, or epigenetic silencing, increases DNA damage tolerance and can be associated with increased TMB (Chalmers et al., 2017). However, no DNA damage/repair-related pathways were found in the results of GO and KEGG enrichment analysis, which indicated that TP53 R273C mutation could affect TMB *via* other pathways. Then, we performed WGCNA on the differential genes between R273C mutation and wild type, trying to find the potential mechanism of TP53 R273C mutation affecting TMB. Finally, 61 genes were identified, which contained 13 HOX family genes. Interestingly, another study also found TMB was related to many HOX genes (Cai et al., 2019). HOX genes are developmental genes—they code for proteins that function as critical master regulatory transcription factors during embryogenesis. Many reports have shown that the protein products of HOX genes also play key roles in the development of cancers (Bhatlekar et al., 2014). Whether TMB is associated with HOX genes needs to be further confirmed.

With the development of immunotherapy, p53 has been regarded as a druggable target because specific TCRs for some of the hotspot mutation of TP53 have been found (Malekzadeh et al., 2019; Malekzadeh et al., 2020). On the other hand, synthetic lethality with p53 is another new potential strategy that makes p53 druggable, which depends on the mutp53 LOFs and GOFs (Jiahao Hu et al., 2021). The present study provides a new insight into the function of the TP53 R273C mutant, which may be beneficial to the treatment of cancer patients in the future.

## Conclusion

In conclusion, we found that the TP53 R273C mutant showed the highest mutation frequency among all the TP53 mutants in LGGs and was associated with poor prognosis, suggesting that TP53 R273C mutation may be oncogenic. Moreover, TP53 R273C mutation may be related to M2 macrophage enrichment and a higher TMB in LGGs.

## Data Availability

RNA-Seq and somatic mutation data were downloaded from the TCGA data portal (https://gdc-portal.nci.nih.gov/). Clinical information was downloaded from cBioPortal (https://www.cbioportal.org/).
