# Supplementary material for: TP53 R273C Mutation Is Associated With Poor Prognosis in LGG Patients
Source: Front Genet. 2022 Mar 11;13:720651. doi: 10.3389/fgene.2022.720651 (PMC8974296; doi:10.3389/fgene.2022.720651)
Supplement: Supplementary file 1 [file DataSheet1.docx]

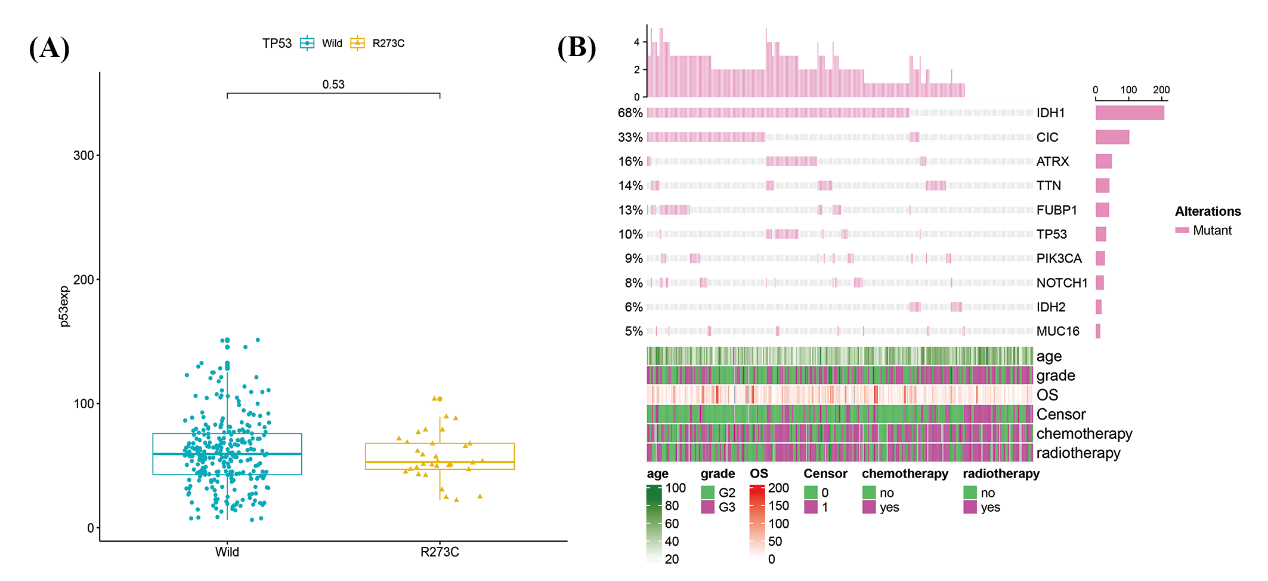


**Fig.S1**.

The mRNA expression level of TP53 and the mutation landscape of LGGs. (A) TP53 expression levels is shown as normalized transcripts per million (TPM). (B) The TP53 mutation only include R273C mutant.


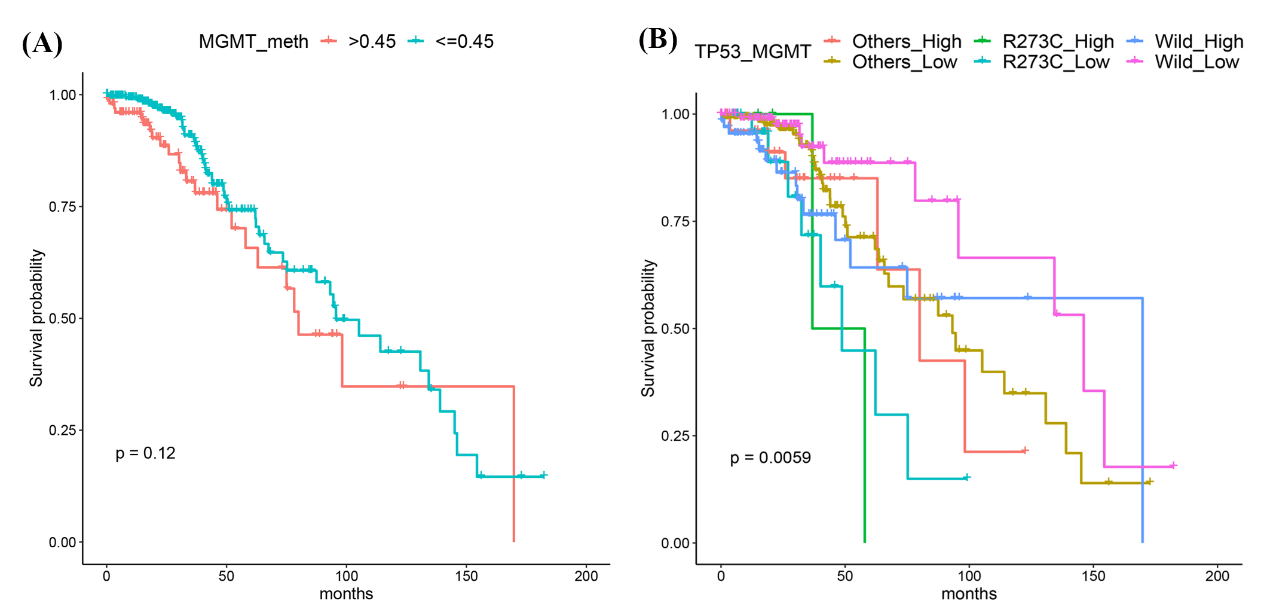


**Fig.S2**.

MGMT promoter methylation and TP53 mutation in LGGs with IDH1/2 mutation. (A) The prognostic significance of MGMT promoter methylation in LGG patients with IDH1/2 mutation. The cut-off value was determined by minimum p-value method. (B) Kaplan-Meier curve grouped by TP53 mutation status and MGMT promoter methylation level.


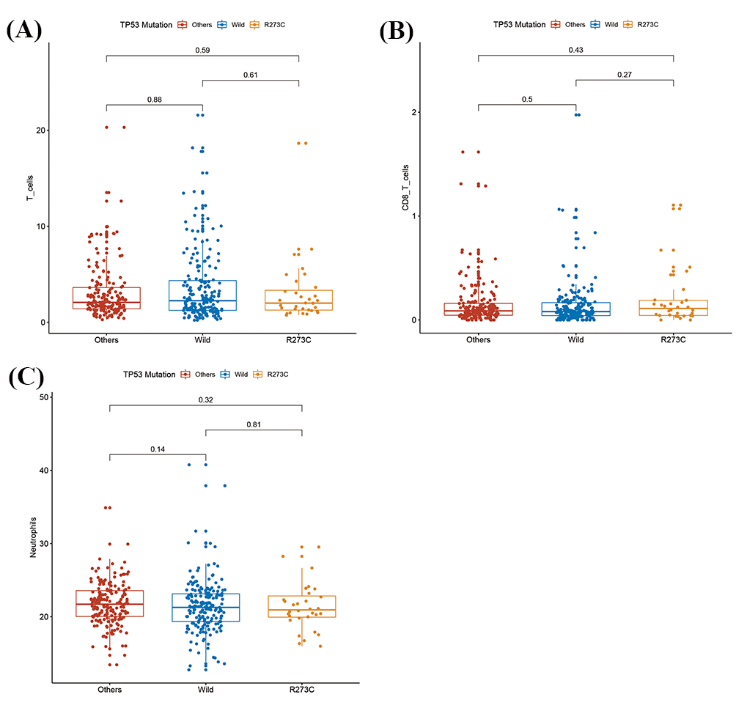


**Fig.S3**.

Immune cell infiltration comparison between different groups. (A) T cells (B) CD8+ T cells (C) neutrophils


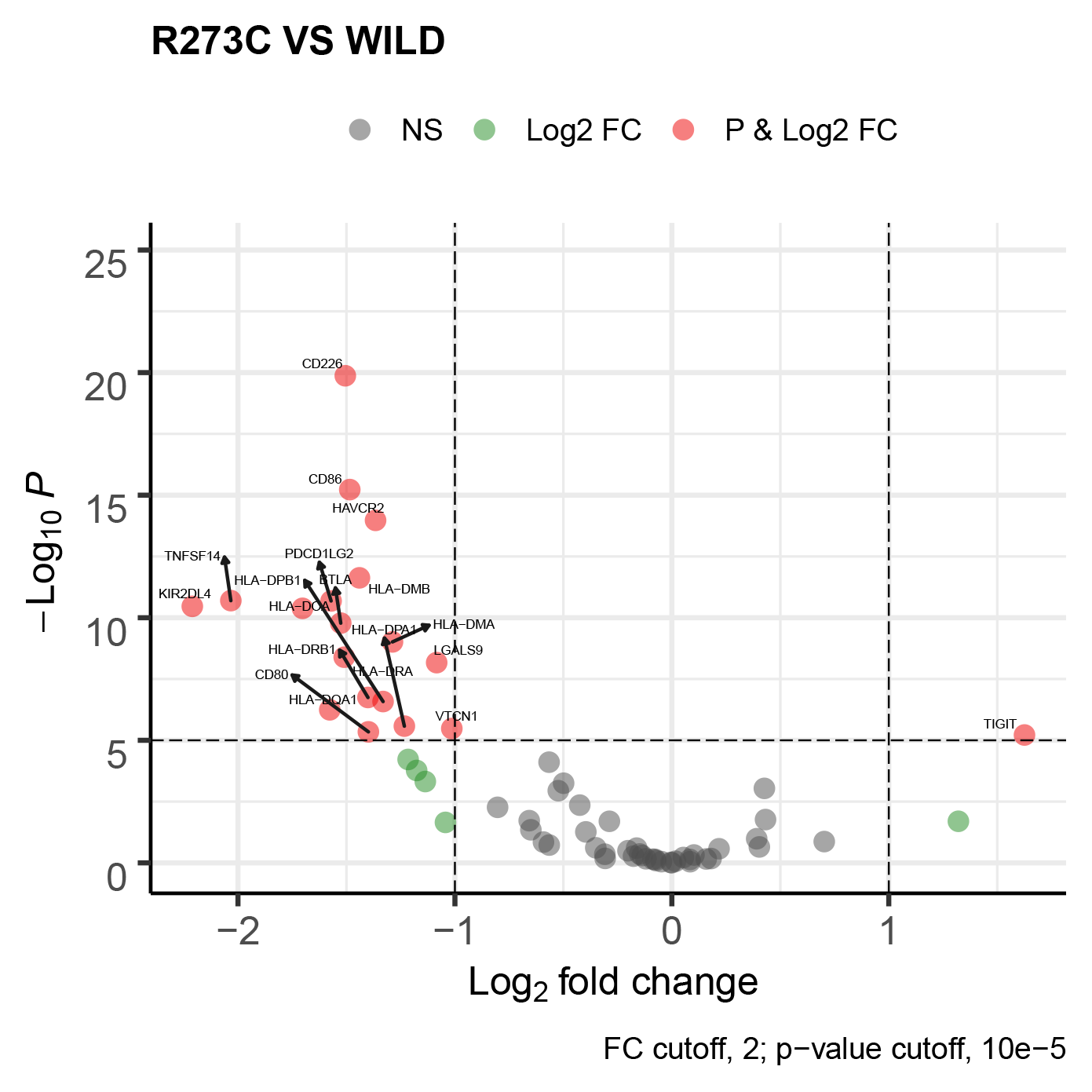


**Fig.S4**.

The difference of expression level of 63 immune checkpoint genes between TP53 mutation status.


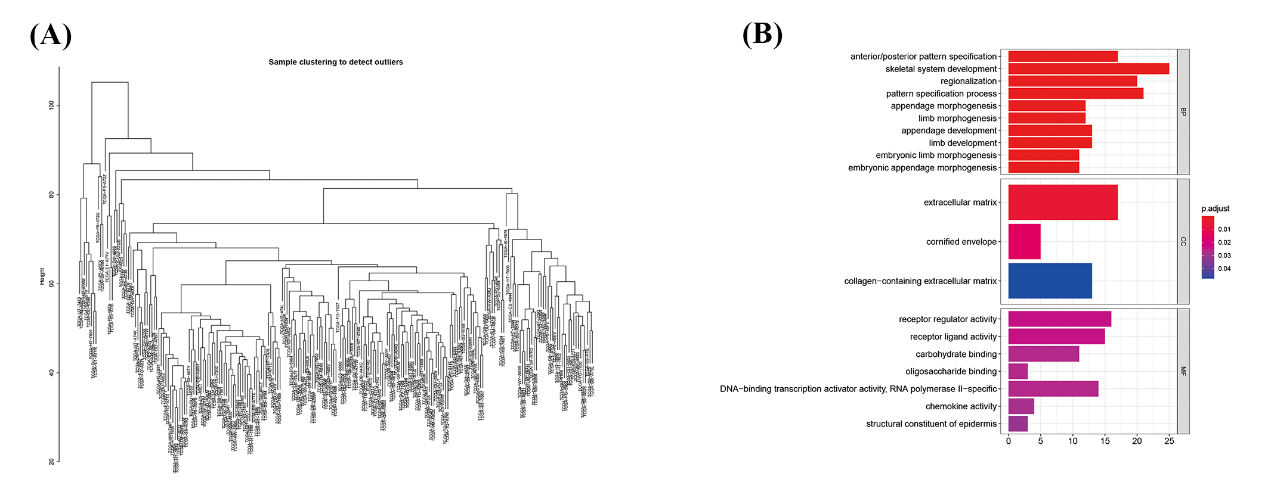


**Fig.S5**

Sample clustering and functional annotation in WGCNA analysis. (A) sample clustering. (B) GO enrichment analysis of grey module.


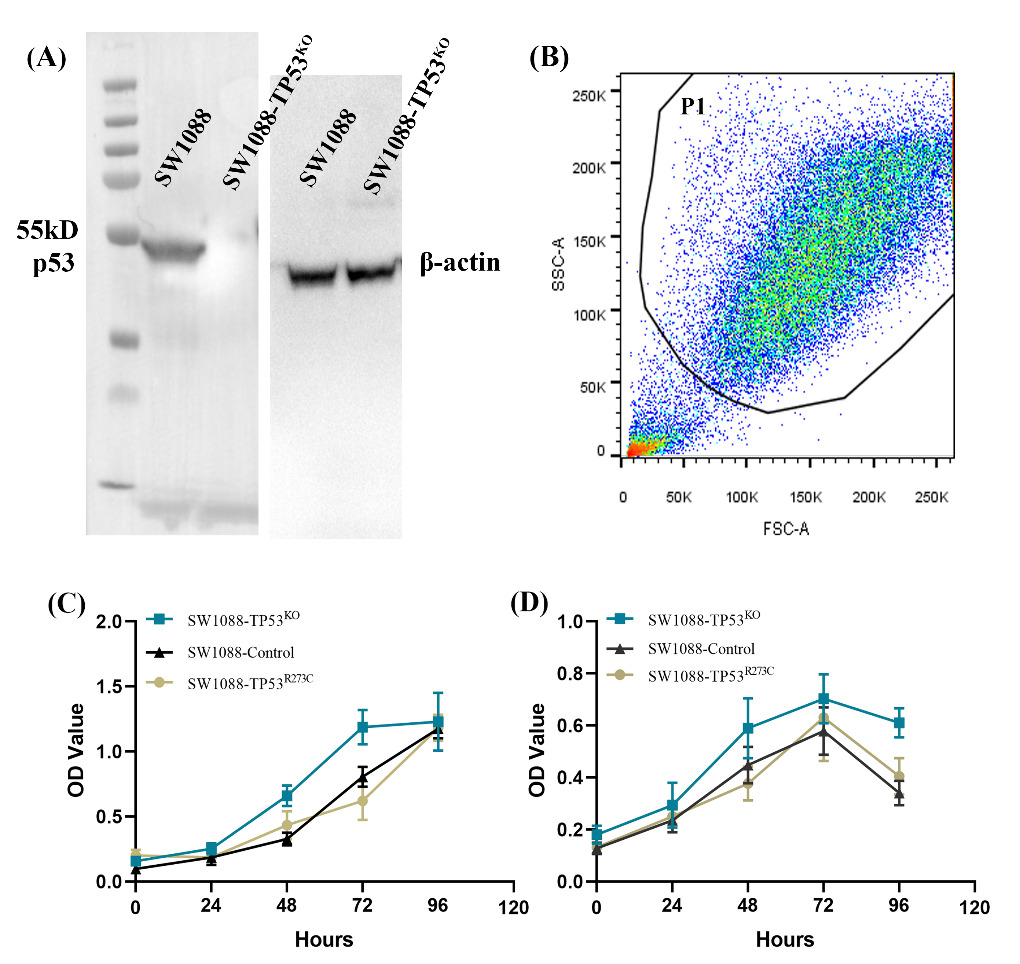


**Fig.S6**

Supplementary material in assays. (A) WB results of TP53 expression in SW1088 and SW1088-TP53KO. (B) An example of gating strategy (FSC vs SSC) for Flow cytometry analysis of apoptosis. (C) Cell proliferation assay using CCK8 (no TMZ). (D) Cell proliferation assay using CCK8 (TMZ 500 μM)
